# Tobacco smoking-attributable mortality in Kenya, 2012–2021

**DOI:** 10.18332/tid/186170

**Published:** 2024-07-24

**Authors:** Lazarus Odeny, Gladwell Gathecha, Valerian Mwenda, Anne Kendagor, Samuel Cheburet, Beatrice Mugi, Caroline Mithi, Florence Jaguga, Kennedy Okinda, Rachel K. Devotsu, Shukri F. Mohamed, Jane Rahedi Ong’ang’o

**Affiliations:** 1 Centre for Respiratory Diseases Research, Kenya Medical Research Institute, Kisumu, Kenya; 2 Division of Non-communicable Diseases, Kenya Ministry of Health, Nairobi, Kenya; 3 Radiology Department, Kenyatta National Hospital, Nairobi, Kenya; 4 Internal Medicine Department, Kenyatta University Teaching, Referral and Research Hospital, Nairobi, Kenya; 5 Alcohol and Drug Abuse Rehabilitation Services Department, Moi Teaching and Referral Hospital, Eldoret, Kenya; 6 Research and Program Department, Kenyatta National Hospital, Othaya, Kenya; 7 Development Gateway, Nairobi, Kenya; 8 Chronic Disease Management Unit, African Population and Health Research Center, Nairobi, Kenya

**Keywords:** tobacco smoking, smoking attributable mortality, population attributable fraction, smoking prevalence, tobacco control

## Abstract

**INTRODUCTION:**

Tobacco smoking poses a significant risk for various diseases, including cardiovascular diseases, chronic respiratory diseases, and cancers. In Kenya, tobacco-related deaths contribute substantially to non-communicable disease mortality. This study aims to quantify the mortality attributed to tobacco smoking in Kenya from 2012 to 2021.

**METHODS:**

Employing a prevalence-based analysis model, the study utilized population attributable fraction (PAF) to estimate age-specific smoke attributable mortality (SAM) rates for individuals aged ≥35 years. Causes of death associated with tobacco use, including cancers, cardiovascular diseases, respiratory diseases, tuberculosis, and diabetes, were analyzed based on age, sex, and death records between 2012 and 2021.

**RESULTS:**

Over the study period, 60228 deaths were attributed to tobacco-related diseases, with an annual increase observed until 2016 and subsequent fluctuations. Respiratory diseases, diabetes mellitus, malignant cancers, tuberculosis, and cardiovascular diseases collectively accounted for 16.5% of deaths among individuals aged ≥35 years. Notable contributors were pneumonia and influenza (respiratory diseases), esophageal cancer (cancers), and cerebrovascular diseases (cardiovascular diseases). Of the observed deaths, 16.5% were attributed to smoking, with respiratory diseases (40.5%), malignant cancers (31.4%), tuberculosis (13%), cardiovascular diseases (8.9%), and diabetes mellitus (6.1%) contributing. Pneumonia and influenza, esophageal cancer, chronic airway obstruction, and tuberculosis were primary causes, comprising 70% of all SAM.

**CONCLUSIONS:**

Tobacco-related mortality is a significant public health concern in Kenya. Efforts should focus on preventing tobacco use and managing associated disease burdens. Smoking cessation initiatives and comprehensive tobacco control measures are imperative to mitigate the impact on population health.

## INTRODUCTION

Tobacco smoking poses a significant risk for a range of illnesses, including cardiovascular diseases (CVD), chronic respiratory conditions, and various cancers affecting organs such as the lungs^1-4^. Globally, this habit contributes to a staggering 7.69 million deaths and 200 million disability-adjusted life-years (DALYs)^5^. In 2019, leading causes of mortality linked to tobacco use were ischemic heart disease (IHD), chronic obstructive pulmonary disease (COPD), tracheal and bronchus cancers, lung cancers, and stroke, collectively responsible for approximately 72% of tobacco-related deaths^5^.

Turning our attention to Kenya, findings from the 2015 STEPs-survey and the Kenya Global Adults Tobacco Survey (GATS)^6-8^ underscore cardiovascular diseases as the primary cause of death associated with tobacco use, followed by cancers, respiratory diseases, and diabetes, accounting for 82% of all non-communicable disease (NCD) deaths linked to tobacco use.

Kenya aligns with the World Health Organization Framework Convention on Tobacco Control (WHO FCTC) to combat the global tobacco epidemic. The focal national policy addressing tobacco use is the Tobacco Control Act of 2007^9^, regulating the manufacturing, advertising, promotion, and sale of tobacco products. Emphasizing smoke-free environments, pictorial health warnings on packaging, and restrictions on advertising and sponsorship, the act prohibits smoking in specific public places, mandates health warnings, and limits tobacco advertising. Oversight and implementation are the responsibilities of the Tobacco Control Board, covering public awareness campaigns, compliance monitoring, and legal actions against violators. Nevertheless, the STEPs-Survey of 2015 and GATS of 2014^6-8^ reported an overall tobacco use prevalence in Kenya of 13.5%, with significantly higher rates among males (23%) compared to females (4%).

Despite the substantial public health impact of tobacco, there is a scarcity of studies investigating the mortality burden of tobacco smoking in Kenya. Notable among these limited studies is a retrospective analysis by Ogeng’o et al.^10^ in 2010, utilizing data from Kenyatta National Hospital, which found a 12.5% increase in the risk of myocardial infarction associated with smoking. Additionally, research by Macigo et al.^11^ revealed that smokers of filter cigarettes had a relative risk of 9.1 for oral leukoplakia, while non-filter cigarette smokers had a risk of 9.8.

Addressing this knowledge gap, our study aims to comprehensively investigate and quantify the mortality attributed to tobacco smoking in Kenya for the period 2012–2021.

## METHODS

### Study design

Our study employed a prevalence-based analysis model, as recommended by Pérez-Ríos and Montes^12^ and Pérez-Ríos et al.^13^, for estimating attributable mortality related to tobacco smoking. This model relies on the population attributable fraction (PAF), quantifying the proportion of deaths in the population attributed to a specific risk factor, such as tobacco smoking. Specifically, it calculates age-specific smoke attributable mortality (SAM) rates for individuals aged ≥35 years, factoring in age, sex, and cause-specific mortality rates. This method, widely acknowledged for SAM calculations, was deliberately chosen due to the absence of cancer mortality data among never smokers in Kenya, ensuring a robust estimation of mortality attributable to tobacco smoking.

The prevalence-based approach proves effective in situations where detailed cancer mortality data for non-smokers are unavailable. By employing this model, we could confidently estimate the mortality linked to tobacco smoking in Kenya. The model’s consideration of age-specific SAM rates, along with demographic and cause-specific factors, enhances the precision of our estimations.

This methodological choice offers notable advantages. Firstly, it allows the computation of SAM rates even when comprehensive cancer mortality data for non-smokers are lacking. Secondly, it adheres to international standards for estimating tobacco-related mortality^13^. Thirdly, it facilitates meaningful comparisons with other studies utilizing similar prevalence-based models, thereby enhancing the generalizability of our findings.

By consciously selecting the prevalence-based analysis model, our objective was to provide a comprehensive and dependable assessment of the mortality burden attributed to tobacco smoking in Kenya, thereby contributing to a broader understanding of the impact of tobacco use on public health.

### Non-communicable disease causes of death

Tobacco use has been linked to non-communicable diseases such as cancers, cardiovascular diseases, chronic respiratory diseases, tuberculosis^14-16^, and diabetes^17^. We adopted a similar disaggregation by sex and age-group in our study. Recognizing that the effects of tobacco use manifest later after smoking initiation, we focused on causes of deaths observed in individuals aged ≥35 years in Kenya between 2012 and 2021. Causes of deaths were stratified into two age groups: 35–64 years and ≥65 years. Additionally, causes of deaths were stratified by sex.

### Observed all-cause mortality

To determine all-cause mortality, we utilized data from the United Nations website^18^ for annual population estimates. Crude death rates for tobacco-related diseases were calculated against national population projections. Joinpoint regression analysis using Joinpoint software 4.9.1.0-April 2022 (Statistic Research and Application Branch, National Cancer Institute) was applied to identify changes in mortality rate trends for every age and sex group. This method determines the year(s) when a trend change occurs based on crude mortality rates. We integrated population data with all-cause mortality data to model deaths attributable to tobacco smoking in Kenya between 2012 and 2021. The mortality data were extracted from the Kenya Health Information System (KHIS), encompassing de-identified, case-based data from health facility-based deaths in Kenya.

All-cause mortality data between 2012 and 2021 were systematically reviewed from hospital medical records. A total of 500 facilities were sampled, including 3 national teaching and referral hospitals and a stratified sample of sub-county and faith-based hospitals. Recertification of the cause of death was performed using WHO-recommended forms, and data were coded using the 10th International Classification of Diseases (ICD-10)^19^.

### Smoking prevalence in Kenya: mid-year analysis

Data on smoking prevalence were obtained from the STEPS survey 2015^6^, a national cross-sectional household survey covering individuals aged 18–69 years. The GATS survey 2014^7^ was also utilized, which used multistage stratified cluster sampling of 5376 households. Both surveys provided insights into tobacco use and measures of interest. Relative risks of death among smokers and ex-smokers compared to non-smokers were derived from recent systematic reviews and the Cancer Prevention Study II of 1982–1988. For our research, we used the relative risks proposed by the Cancer Prevention Study II and the Royal College of Physicians in 2020^20^.

### Calculation of smoking attributable mortality

The study calculated SAM for each cause of mortality using the formula:

SAM = OM × PAF

where OM represents observed mortality, and PAF is the population attributable fraction. PAF was determined by the formula:

PAF = [(p0 + p1×RR1 + p2×RR2) – 1]/(p0 + p1×RR1 + p2×RR2)

with p0, p1, and p2 representing the prevalence of non-smokers, current smokers, and former smokers, respectively, and RR1 and RR2 are the risks of dying from any cause for current smokers and ex-smokers, respectively. The SAM formula encapsulated a comprehensive estimation of mortality associated with tobacco smoking, considering the prevalence and risks of different smoking categories. The calculation allowed for the determination of the proportion of deaths attributed to smoking, providing a quantifiable measure of the mortality burden associated with tobacco smoking in the studied population. The methodology integrated data from multiple sources and employed statistical analysis techniques to estimate tobacco-related mortality in Kenya, considering age, sex, and specific causes of death. Ethical considerations, data reliability, and the use of established models contributed to the robustness of the study’s methodology^12,13,21^.

### Ethical approval

Ethical approval was not sought since our study utilized data from existing medical records rather than human subjects. However, administrative approval was obtained from the Principal Secretary of the Ministry of Health, the Director of Civil Registration Services, and County Directors of Health to permit the use of records.

## RESULTS

### Observed mortality

**Figure 1:**
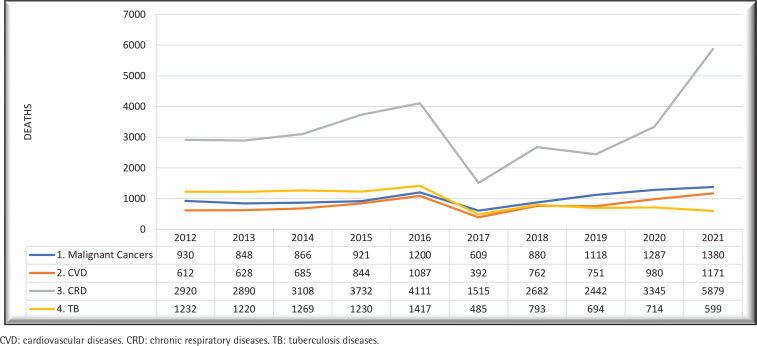
Absolute mortality numbers from medical records of persons aged **≥**35 years who died due to smoking-related diseases in Kenya, 2012–2021

**Figure 2:**
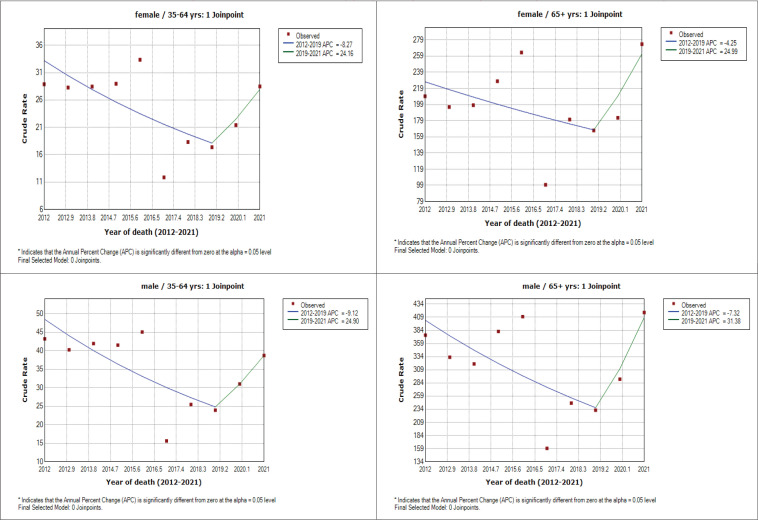
Mortality rate trends for 60228 persons aged **≥**35 years who died due to tobacco-related diseases in Kenya, 2012–2021

Between 2012 and 2021, Kenya experienced 60228 deaths attributed to tobacco-related diseases among adults aged ≥35 years. Median age was 65 years (IQR: 50–77). Males accounted for the majority at 56% (Table 1). The observed mortality demonstrated an annual increase across all cohorts (males and females 35–64 years and ≥65 years) until 2016, with a notable decline in 2017 due to a health-workers strike affecting record-keeping. Subsequently, mortality rates fluctuated, reaching a sharp increase after 2018 (Figures 1 and 2). Joinpoint regression analysis revealed a decline in mortality rates until 2018, followed by a substantial rise.

**Table 1:** Absolute numbers of recorded deaths of persons aged ≥35 years who died due to tobacco related diseases in Kenya, 2012–2021

*Tobacco-related diseases*	*2012 n (%)*	*2013 n (%)*	*2014 n (%)*	*2015 n (%)*	*2016 n (%)*	*2017 n (%)*	*2018 n (%)*	*2019 n (%)*	*2020 n (%)*	*2021 n (%)*	*Total n (%)*
**1. Malignant cancers (Cancer)**	930 (16)*	848 (15)	866 (15)	921 (14)	1200 (15)	609 (20)	880 (17)	1118 (22)	1287 (20)	1380 (15)	10039 (17)
Esophagus C15	326 (35)**	300 (35)	344 (40)	346 (38)	484 (40)	240 (39)	351 (40)	429 (38)	441 (34)	438 (32)	3699 (37)
Kidney and renal pelvis C64-C65	6 (1)	5 (1)	7 (1)	11 (1)	6 (1)	5 (1)	9 (1)	12 (1)	12 (1)	17 (1)	90 (1)
Larynx C32	17 (2)	11 (1)	10 (1)	12 (1)	16 (1)	10 (2)	6 (1)	18 (2)	21 (2)	49 (4)	170 (2)
Lips, oral cavity, pharynx C00–C14	41 (4)	47 (6)	48 (6)	55 (6)	55 (5)	48 (8)	53 (6)	60 (5)	105 (8)	122 (9)	634 (6)
The neck of the uterus C53	186 (20)	187 (22)	177 (20)	163 (18)	224 (19)	115 (19)	170 (19)	179 (16)	236 (18)	277 (20)	1914 (19)
Pancreas C25	94 (10)	84 (10)	58 (7)	79 (9)	105 (9)	37 (6)	81 (9)	94 (8)	122 (9)	135 (10)	889 (9)
Stomach C16	178 (19)	150 (18)	150 (17)	169 (18)	195 (16)	102 (17)	122 (14)	203 (18)	186 (14)	196 (14)	1651 (16)
Trachea, lungs, bronchi C33–C34	52 (6)	41 (5)	44 (5)	55 (6)	79 (7)	34 (6)	73 (8)	98 (9)	136 (11)	118 (9)	730 (7)
Urinary bladder C67	30 (3)	23 (3)	28 (3)	31 (3)	36 (3)	18 (3)	15 (2)	25 (2)	28 (2)	28 (2)	262 (3)
**2. Cardiovascular diseases (CVD)**	612 (11)	628 (11)	685 (12)	844 (13)	1087 (14)	392 (13)	762 (15)	751 (15)	980 (15)	1171 (13)	7912 (13)
Cerebrovascular disease I60–I69	518 (85)	570 (91)	609 (89)	735 (87)	968 (89)	343 (88)	657 (86)	647 (86)	826 (84)	997 (85)	6870 (87)
Ischemic heart disease I20-I25	70 (11)	46 (7)	63 (9)	91 (11)	92 (8)	37 (9)	77 (10)	74 (10)	118 (12)	131 (11)	799 (10)
Other arterial diseases I72-I78	24 (4)	12 (2)	13 (2)	18 (2)	27 (2)	12 (3)	28 (4)	30 (4)	36 (4)	43 (4)	243 (3)
**3. Respiratory diseases (CRD)**	1953 (34)	1943 (35)	2122 (36)	2485 (37)	2794 (36)	977 (33)	1698 (33)	1553 (31)	2071 (33)	4075 (45)	21671 (36)
Bronchitis, emphysema J40-J43	19 (1)	14 (1)	23 (1)	16 (1)	23 (1)	8 (1)	6 (0)	12 (1)	11 (1)	13 (0)	145 (1)
Chronic airway obstruction J44–J46	290 (15)	279 (14)	260 (12)	313 (13)	409 (15)	155 (16)	256 (15)	225 (14)	268 (13)	362 (9)	2817 (13)
Pneumonia, Influenza J10-J18	1644 (84)	1650 (85)	1839 (87)	2156 (87)	2362 (85)	814 (83)	1436 (85)	1316 (85)	1792 (87)	3700 (91)	18709 (86)
**4. Tuberculosis (TB)**	1232 (22)	1220 (22)	1269 (21)	1230 (18)	1417 (18)	485 (16)	793 (15)	694 (14)	714 (11)	599 (7)	9653 (16)
**5. Diabetes mellitus**	967 (17)	947 (17)	986 (17)	1247 (19)	1317 (17)	538 (18)	984 (19)	889 (18)	1274 (20)	1804 (20)	10953 (18)
**Total**	5694 (100)	5586 (100)	5928 (100)	6727 (100)	7815 (100)	3001 (100)	5117 (100)	5005 (100)	6326 (100)	9029 (100)	60228 (100)

* Percentage is of subgroup over total deaths (i.e. 16% cancer deaths in the year 2012 = 930/5694).

** Disease specific percentage is over the subgroup [i.e. 35% of esophagus C15 deaths for year 2012 = esophagus C15 deaths over all cancer deaths (326/930)]. CRD: chronic respiratory diseases.

The major contributors to mortality included respiratory diseases (36%), diabetes mellitus (18%), malignant cancers (17%), tuberculosis (16%), and cardiovascular diseases (13%). Among respiratory infections, pneumonia and influenza (86%) predominated, surpassing COPD (13%) and bronchitis/emphysema (1%). The year 2020 witnessed a significant two-fold increase in pneumonia and influenza deaths, attributed to COVID-19. Noteworthy cancer causes included esophagus cancer (37%), cervical cancer (19%), stomach cancer (16%), and pancreatic cancer (9%). Cerebrovascular diseases (87%) emerged as the primary cardiovascular cause of death, followed by ischemic heart diseases (10%) and other arterial diseases (3%) (Table 1).

### Smoking prevalence

Mid-decade prevalence of tobacco smoking (2012–2021), including persons aged <35 years, indicated that 17.4% of men, 0.9% of women, and 9% overall were current smokers; former smokers constituted 10.6% of men, 1.4% of women, and 5.9% overall. Among individuals aged ≥35 years (our study population), 24.1% of men, 1.4% of women, and 12.9% overall were current smokers, while 17.3% of men, 2.4% of women, and 10% overall were former smokers (Table 2).

**Table 2:** Smoking mid-decade (2012–2021) prevalence estimates in Kenya, by gender and age

	*Proportion of smokers and non-smokers by smoking category (row%)*		
	*Current*	*Former*	*Never*
**Total**	12.9	10.0	77.2
Age 35–64 years	13.5	9.4	77.2
Age ≥65 years	8.1	14.0	78.0
**Male**	24.1	17.3	58.7
Age 35–64 years	24.8	16.3	59.0
Age ≥65 years	18.5	24.8	56.7
**Female**	1.4	2.4	96.3
Age 35–64 years	1.5	1.9	96.7
Age ≥65 years	0.7	6.1	93.3

### Smoking-attributable mortality

Out of the 60228 observed deaths from respiratory diseases, diabetes mellitus, malignant cancers, tuberculosis, and cardiovascular diseases between 2012 and 2021, 16.5% (9943) were attributed to tobacco smoking (Table 3). This included 40.5% from respiratory diseases, 31.4% from malignant cancers, 13% from tuberculosis, 8.9% from cardiovascular diseases, and 6.1% from diabetes mellitus. Within respiratory diseases, pneumonia and influenza contributed 59%, while chronic airway obstruction accounted for 39%. Noteworthy cancer causes included esophageal cancer (56%) and trachea, lungs, and bronchi combined cancers (14%). Of the cardiovascular tobacco-attributable deaths, 83% were from cerebrovascular diseases, with a notable distribution of 58% and 25% among 35–64 years and ≥65 years, respectively. The primary causes for smoking-attributable deaths were pneumonia and influenza (24%), esophageal cancer (18%), chronic airway obstruction (16%), and tuberculosis (13%), constituting 70% of all SAM (6987/9943).

**Table 3:** Tobacco smoking attributable deaths of persons aged ≥35 years who died due to tobacco-related diseases in Kenya, 2012–2021

*TRI and ICD10 codes*	*Observed mortality*			*Age-adjusted relative risk CPS-II (1982–1988)*				*Prevalence estimate (mid-decade 2012–2021 GATS & STEPS)*						*PAR*			*Smoking attributable mortality (SAM)*			
	*Female*	*Male*	*Total*	*Female*		*Male*		*Female*			*Male*			*Female*	*Male*	*Total*	*Female*	*Male*	*Total*	*Cause specific*
				*CS*	*FS*	*CS*	*FS*	*CS*	*FS*	*Never*	*CS*	*FS*	*Never*							
	*n (%)*	*n (%)*	*n*	*RR*	*RR*	*RR*	*RR*	*%*	*%*	*%*	*%*	*%*	*%*	*%*	*%*	*%*	*n (%)*	*n (%)*	*n*	*%*
**1. Malignant cancers (Cancer)**	4990 (49.7)	5049 (50.3)	10039													31.1	279 (8.9)	2847 (91.1)	3126	31.4
Esophagus C15	1288 (34.8)	2411 (65.2)	3699	7.8	2.8	6.8	4.5	1.4	2.4	96.3	24.05	17.3	58.7	11.8	66.7	47.6	152 (8.7)	1607 (91.3)	1759	17.7
Kidney and renal pelvis C64-C65	46 (51.1)	44 (48.9)	90	1.3	1.1	2.7	1.7	1.4	2.4	96.3	24.05	17.3	58.7	0.6	34.6	17.3	0 (1.9)	15 (98.1)	16	0.2
Larynx C32	29 (17.1)	141 (82.9)	170	13	5.2	14.6	6.3	1.4	2.4	96.3	24.05	17.3	58.7	20.7	80.7	70.5	6 (5.0)	114 (95.0)	120	1.2
Lips, oral cavity, pharynx C00–C14	223 (35.2)	411 (64.8)	634	5.1	2.3	10.9	3.4	1.4	2.4	96.3	24.05	17.3	58.7	7.9	73.6	50.5	18 (5.5)	303 (94.5)	320	3.2
The neck of the uterus C53	1914 (100.0)	0 (0.0)	1914	1.6	1.1			1.4	2.4	96.3				1.0		1.0	20 (100.0)	0 (0.0)	20	0.2
Pancreas C25	463 (52.1)	426 (47.9)	889	2.3	1.6	2.3	1.2	1.4	2.4	96.3	24.05	17.3	58.7	3.1	25.8	13.9	14 (11.5)	110 (88.5)	124	1.2
Stomach C16	637 (38.6)	1014 (61.4)	1651	1.4	1.3	2	1.5	1.4	2.4	96.3	24.05	17.3	58.7	1.2	24.6	15.6	8 (3.0)	250 (97.0)	258	2.6
Trachea, lungs, bronchi C33–C34	296 (40.5)	434 (59.5)	730	12.7	4.5	23.3	8.7	1.4	2.4	96.3	24.05	17.3	58.7	19.4	87.0	59.6	57 (13.2)	378 (86.8)	435	4.4
Urinary bladder C67	94 (35.9)	168 (64.1)	262	2.2	1.9	3.3	2.1	1.4	2.4	96.3	24.05	17.3	58.7	3.6	42.6	28.6	3 (4.5)	72 (95.5)	75	0.8
**2. Cardiovascular diseases (CVD)**	3907 (49.4)	4005 (50.6)	7912													11.2	66 (7.4)	818 (92.6)	884	8.9
Cerebrovascular disease I60–I69 (35–64 years)	1026 (44.4)	1283 (55.6)	2309	4	1.3	3.3	1	1.5	1.9	96.65	24.8	16.3	58.95	4.7	36.3	22.3	48 (9.4)	466 (90.6)	514	5.2
Cerebrovascular disease I60–I69 (≥65 years)	2431 (53.3)	2130 (46.7)	4561	1.5	1	1.6	1	0.7	6.1	93.25	18.5	24.8	56.7	0.3	10.0	4.8	8 (3.6)	213 (96.4)	221	2.2
Ischemic heart disease (IHD) I20-I25 (35–64 years)	126 (34.7)	237 (65.3)	363	3.1	1.3	2.8	1.6	1.5	1.9	96.65	24.8	16.3	58.95	3.5	35.2	24.2	4 (5.0)	83 (95.0)	88	0.9
Ischemic heart disease (IHD) I20-I25 (≥65 years)	218 (50.0)	218 (50.0)	436	1.6	1.2	1.5	1.2	0.7	6.1	93.25	18.5	24.8	56.7	1.6	12.4	7.0	3 (11.3)	27 (88.7)	31	0.3
Other arterial disease I72-I78	106 (43.6)	137 (56.4)	243	2.2	1.1	2.1	1	1.4	2.4	96.3	24.05	17.3	58.7	1.8	20.9	12.6	2 (6.3)	29 (93.7)	31	0.3
**3. Respiratory diseases (CRD)**	9178 (42.4)	12493 (57.6)	21671													18.6	413 (10.2)	3617 (89.8)	4030	40.5
Bronchitis, emphysema J40-J43	46 (31.7)	99 (68.3)	145	12	11.8	17.1	15.6	1.4	2.4	96.3	24.05	17.3	58.7	28.7	86.5	68.1	13 (13.4)	86 (86.6)	99	1.0
Chronic airway obstruction J44–J46*	1099 (39.0)	1718 (61.0)	2817	13.1	6.8	10.6	6.8	1.4	2.4	96.3	24.05	17.3	58.7	23.1	76.8	55.8	253 (16.1)	1319 (83.9)	1573	15.8
Pneumonia, influenza J10-J18	8033 (42.9)	10676 (57.1)	18709	2.2	1.1	1.8	1.4	1.4	2.4	96.3	24.05	17.3	58.7	1.8	20.7	12.6	146 (6.2)	2212 (93.8)	2359	23.7
**4. Diabetes mellitus**	5428 (49.6)	5525 (50.4)	10953	1.37	1.14	1.37	1.14	1.4	2.4	96.3	24.05	17.3	58.7	0.8	10.2	5.5	45 (7.4)	562 (92.6)	606	6.1
5. Tuberculosis (TB)*	3194 (33.1)	6459 (66.9)	9653	1.57	1.57	1.57	1.57	1.4	2.4	96.3	24.05	17.3	58.7	2.1	19.1	13.4	66 (5.1)	1231 (94.9)	1297	13.0
**All deaths**	26697 (44.3)	33531 (55.7)	60228													16.5	868 (8.7)	9075 (91.3)	9943	100

* SAM calculated based on p0, p1, and p2 representing the prevalence of non-smokers, current smokers, and former smokers, respectively. CS: current smoker. FS: former smoker. CPS-II: The 2nd Cancer Prevention Study. PAR: population attributable risk. CRD: chronic respiratory diseases.

## DISCUSSION

### Magnitude of smoking-attributable mortality

In this study, the examination of all-cause mortality data between 2012 and 2021 revealed a substantial impact of smoking, contributing to 16.5% of deaths among adults aged ≥35 years in Kenya. These findings align with a growing body of evidence underscoring the profound health implications associated with smoking^20^.

### Trends in mortality and global comparisons

The observed trend of escalating mortality across the studied conditions throughout the decade mirrors patterns observed in analogous studies. Comparable trends were noted in a study in China examining cancer mortality attributable to tobacco smoking over a ten-year period^22^. However, disparities emerge when comparing our findings with a study in Morocco^23^, where 9.7% of all deaths were attributed to tobacco smoking, notably lower than the 16.5% observed in Kenya.

### Disease-specific contributions to smoking-attributable deaths

*Respiratory diseases*

Respiratory diseases, particularly pneumonia and influenza, emerged as the predominant contributors to deaths attributable to tobacco smoking, followed closely by malignant cancers. Although local data on lung cancer were limited, the similarities with Australia’s 2018 observations reinforce the consistency of these findings^11,24^.

*Chronic respiratory diseases*

Chronic respiratory diseases demonstrated a distinctive pattern in our study, with pneumonia and influenza overshadowing chronic obstructive pulmonary disease (COPD). This contrasts with the Global Burden of Disease (GBD) Study 2019^5^, which highlighted COPD as the primary cause of death in chronic respiratory diseases (CRDs).

*Cancers*

Cancer-related deaths attributed to smoking revealed esophageal cancer as the leading cause, consistent with Chinese findings where lung, liver, esophageal, and stomach cancers were frequently associated with smoking-associated cancer mortality^25,26^.

*Cardiovascular diseases*

Within cardiovascular deaths, cerebrovascular diseases dominated among men, followed by ischemic heart diseases, aligning with established evidence of tobacco smoking’s pervasive impact across the cardiovascular system^27^. Males exhibited a higher risk of cardiovascular disease (CVD) mortality than females.

*Diabetes*

The study also revealed the association between smoking and diabetes-related mortality, consistent with existing epidemiological studies^28^. This underscores the importance of addressing smoking cessation as a crucial aspect of managing diabetes-associated mortality risks^29-31^.

*Tuberculosis*

Moreover, our study illuminated the role of smoking in tuberculosis (TB) mortality in Kenya. While our findings are consistent with the meta-analysis of Bates et al.^14^, attributing 31% of TB cases and deaths to smoking, the percentage in our study was lower (13.4%). The gender disparity in TB deaths attributable to smoking mirrored findings from South Korea, India, and Bangladesh^32-35^, with higher proportions in males.

### Strengths and limitations

The study utilized nationally representative datasets that directly measured smoking status to determine tobacco smoking prevalence averaged over a decade. This robust approach provides a comprehensive overview despite potential fluctuations in smoking prevalence due to various interventions. The adoption of revised relative risks from recent systematic reviews and meta-analyses enhances the accuracy of the prevalence-based model, overcoming previous inconsistencies.

Despite these valuable insights, the study has limitations. It focused exclusively on smoking, neglecting other forms of tobacco use, such as smokeless tobacco, which may contribute significantly to morbidity and mortality. The challenge of ill-defined causes of death and the need for improved death certification processes highlight the study’s limitations. Efforts to enhance the quality of cause-of-death certification, including periodic reviews and training for healthcare providers, are imperative.

## CONCLUSIONS

Our study establishes smoking-attributable mortality as a critical health concern in Kenya. Urgent and concerted efforts are needed to prevent tobacco use and address the associated disease burden. Immediate tobacco control imperatives should focus on facilitating smoking cessation among existing smokers. Continuous monitoring, public awareness campaigns, and targeted interventions are vital components of a comprehensive strategy to mitigate the impact of smoking-attributable deaths in Kenya.

## Data Availability

The data supporting this research are available from the authors on reasonable request.
